# Socio-Emotional and Cognitive Development in Intrauterine Growth Restricted (IUGR) and Typical Development Infants: Early Interactive Patterns and Underlying Neural Correlates. Rationale and Methods of the Study

**DOI:** 10.3389/fnbeh.2018.00315

**Published:** 2018-12-18

**Authors:** Chiara Sacchi, Pietro De Carli, Giovanni Mento, Teresa Farroni, Silvia Visentin, Alessandra Simonelli

**Affiliations:** ^1^Department of Developmental Psychology and Socialization, University of Padova, Padova, Italy; ^2^Department of General Psychology, University of Padova, Padova, Italy; ^3^Department of Women’s and Child’s Health, University of Padova, Padova, Italy

**Keywords:** intrauterine stress, fetal growth restriction, face processing, socio-cognitive development, mother-child interactions

## Abstract

Intrauterine growth restriction (IUGR) is defined as a fetal growth retardation, resulting in an estimated fetal weight less than the 10th centile for gestational age. IUGR developing brain is affected by the atypical fetal growth, presenting altered structure and connectivity and increased risk for neurodevelopmental impairments. Behaviorally, IUGR infants show reduced responsiveness and engagement with human faces during mother-child exchanges. The neural mechanisms of these patterns of interactions remain unexplored, as well as their potential role in shaping socio-cognitive trajectories of development. Aim of this research project will be to longitudinally investigate mother-infant interactions and infant’s event-related potential (ERP) components of face processing (infant N170, P400, Negative central) in 4 and 9 months IUGR as potential early markers of expected atypical cognitive and behavioral outcomes observed at 12 months. Thirty IUGR participants will be recruited after receiving the *in utero* diagnosis (>28th gestational week). Thirty healthy infants will be enrolled as the control group. Maternal environment will be assessed via Emotional Availability Scales (EASs), with child responsiveness and maternal sensitivity as variables of interest. Infants’ scalp-recorded cortical activity in response to social and non-social stimuli will be investigated using a high-density EEG system (EGI Geodesic system). Neurodevelopment will be measured at 12 months of child’s life, using Bayley Scales for Infant Development (BSID), while the possible presence of emotional-behavioral problems will be rated via Child Behavior Checklist (CBCL). We expect that being IUGR significantly affects cognitive and behavioral outcomes, through mediation effects of both infants’ neural and behavioral capacity to respond to social stimuli. Indeed, we expect an altered response to social stimuli in IUGR infants, resulting in smaller ERP components amplitude in response to human faces compared to healthy matched peers. A significant association between neural response to social stimuli and infants’ responsiveness to maternal stimulation during interactions is expected, with impoverished performances on the interactive domain in IUGR, compared to healthy peers. This study will enhance understanding on neural mechanisms underpinning the interactive patterns sustaining socio-cognitive development in IUGR and healthy infants. The study will help in clarifying the role of postnatal environment in buffering the vulnerability experienced by children delayed in their fetal growth.

## Introduction

Intrauterine growth restriction (IUGR) is defined as a fetal growth retardation, resulting in an estimated fetal weight (postnatally confirmed by birth weight) on the lowest 10th percentile for gestational age (Alfirevic and Neilson, 1993). By affecting 5% to 7% of pregnancies, IUGR is the second leading cause of perinatal mortality and morbidity worldwide, representing a major public health problem (Murray et al., 2015). A fetal growth unable to reach its genetic potential is a risk factor for later neurodevelopmental outcomes (Kok et al., 2007; Baschat, 2011). In fact, functional impairments have been observed at birth and early in life; specifically, immature attention-interaction scores and impaired visual recognition memory performances are described in 7-month-old infants (Gotlieb et al., 1988; Tolsa et al., 2004), while at 1 year of life, significantly lower scores on Bayley Scales are reported (Fernandez-Carrocera et al., 2003; Batalle et al., 2013). Moreover, growth-restricted infants show poor use of environmental stimuli, reduced social responsiveness, more insulated cry states, and poor motor performance as compared with normal birth weight infants (Padilla et al., 2011). Persisting and long-term outcomes are also observed, with cognitive impairments (e.g., executive functioning; Geva et al., 2006) and behavioral problems described in childhood (Sung et al., 1993); motor problems, learning difficulties and lower academic achievements during school age period (Leitner et al., 2007; Esteban et al., 2010), as well as increased risk for neurodevelopmental disorders, such as ADHD (Heinonen et al., 2013). Apart from evidence of neurodevelopmental and cognitive outcomes, socio-emotional development still appears as unexplored in the developmental context of growth restriction, although few signs of early atypical social interactions are described (Watt, 1990; Feldman and Eidelman, 2009), as well as later poor socio-cognitive performances at school age and mood disorders (Fischi-Gómez et al., 2015).

Literature evidenced several structural and functional brain abnormalities potentially linking fetal growth rate to the detrimental neurodevelopmental and socio-cognitive outcomes. Indeed, IUGR infants show reduced brain volumes as well as delayed and diminished myelination (Dubois et al., 2008; Padilla et al., 2011; Ramenghi et al., 2011). The alterations seem to persist in long term deficits, since motor and cortico-striatal-thalamic networks impairments are observed in 6 years old IUGR children, and delayed myelination as well as disrupted white matter integrity last up to adulthood (Fischi-Gómez et al., 2015). Despite this evidence, the early neural mechanisms sustaining the socio-emotional competencies and the socio-cognitive development in IUGR infants are still underexplored. However, it is of the highest importance to provide comprehension on early markers, both in terms of behavioral and brain mechanisms, of the developmental cascade that begins with early fetal abnormal experience and might potentially result in socio-emotional difficulties and neurodevelopmental outcomes observed later in life. Indeed, in a preventive perspective, targeting a potential early vulnerability in IUGR development, particularly when born at term, could be highly convenient and rewarding. Despite literature extensively reports altered quality in antenatal environment, quite few studies investigated IUGR developmental trajectories, thus neglecting the opportunity to tailor interventions and to develop *ad hoc* follow up mental health care. In addition, the urgency for identifying potential targets for interventions should consider a multifaceted approach, where infant and caregiver are parts of a mutually influencing complex and interrelated system (Sacchi et al., 2018). Taking into account infant and mother’s variables, different potential mechanisms to target could arise from this study. First, detecting vulnerability in processing social stimuli might guide behavioral intervention sustaining parenting abilities to use multi-modal channels of stimulations during interactions. Second, fostering a protective effect of parenting behavior on brain functionality would potentially have an effect on infant socio-emotional development, hopefully compensating for the suspected reduced early communication abilities of IUGR infants. Third, a longitudinal investigation would allow to study different potential windows of plasticity both for typical and atypical development. This could lead to more focused interventions aware of the most susceptible periods and the most rewarding processes to target.

With this theoretical and clinical perspective in mind, we propose a longitudinal investigation of two interrelated mechanisms that might be detected across the first year of life as early markers of potential atypical socio-emotional and cognitive outcomes of IUGR developmental trajectories: infant behavioral responsiveness in social interaction and infant early neural face processing.

### Infant Behavioral Responsiveness in Social Interaction

Within the first year of life, typically developing infants display an amazingly sophisticated set of social behaviors, which foster learning processes in a broad collection of developmental domains (McDonald and Perdue, 2018). These socio-emotional competencies involve the abilities to interact, communicate and deal with emotions, which are primarily experienced in early interactive exchanges with the mother (Bowlby, 1978). Within this affectionate bond, the child receives not only protection, care and the recognition of his/her needs, but also an encompassing environment for physical, cognitive, social and affective development (Britto et al., 2017). Indeed, the mutuality of exchanges between mother and child represents not only a source of stimuli for the child but also an environment sensitive to activities and modifications (van den Bloom and Hoeksma, 1994). In the case of atypical development, infant characteristics can deeply expose the quality of mother child interactions (Kiff et al., 2011). Indeed, the few available studies evidenced that IUGR infants are likely to display difficulties in orientating to social and non-social environment (Watt, 1990) and tend to look at people less frequently than age-matched healthy infants; also, higher levels of negative affect are reported, evidencing an early vulnerability in communication skills (Watt and Strongman, 1985; Watt, 1987). As regards, interactive abilities, the very limited findings on IUGR or small for gestational age (SGA) children suggest those infants are more passive during mother-child interactive exchanges, smiling and looking at their mothers’ face less than normal birth weight matched newborns, being less rhythmic and synchronous in daily interactions (Feldman and Eidelman, 2006), and thus appearing as less rewarding interactive partners. A similar interactive pattern is displayed in preterm infants and their mothers, where a scarcity of communicative signals on infant’s side could activate some compensatory behaviors in parents (Miles and Holditch-Davis, 1995; Montirosso et al., 2017). This parenting response can be highly adaptive but can eventually result into intrusive and non-attuned behaviors (Howe et al., 2016). Therefore, on the one hand, atypical development in the domain of diminished early communicative abilities can disrupt mother child exchanges leading to an additional impoverishment of infant’s environment. On the other hand, the quality of mother-child interactions can potentially buffer the negative effect of infant scarce interactive abilities on child development (Baker et al., 2007). In fact, some evidences show the moderating role of maternal sensitivity on infant developmental trajectories. More specifically, recent evidences show the role of mother child interactions on infant’s brain functionality, confirming the relevance of considering mother and child as a broad interrelated system where infant development takes place.

### Infant Neural Face Processing

Among early neural competences displayed by newborn and infants, the ability to recognize and direct the attention to faces appear as highly relevant for socio-cognitive development. Face perception represents an experience-expectant and activity-dependent function (Nelson, 2001; Young et al., 2017); that is critical in the development of higher level social and cognitive functions (Parker and Nelson, 2005). Indeed, the human face provides the infant with a wealth of socially and affectively relevant information and humans appear to be inherently interested in faces, displaying from infancy a strong interest in facial-like figures (Johnson, 1991; Morton and Johnson, 1991). Early disruption or delay in this low-level process can negatively impact infant’s ability to interact with the social environment (Elsabbagh et al., 2015) and disturb natural mutuality in social interaction with potential detrimental effects for child development (Wan et al., 2013). Indeed, huge part of early interactive exchanges rely on the use of face and facial expression are early used to understand others emotion and thought, to make others understand themselves, and to share emotional states (Beebe et al., 2010, 2016). Studies observed that different early stressors and risk factors, such as prolonged institutionalization or risk for autism, are likely to affect this infant capacity that is considered a strong candidate for being one of the mechanisms of the association between early stress and socio-emotional difficulties (Nelson and McCleery, 2008). More specifically, Parker and Nelson (2005) found that the amplitude of the event-related potential (ERP) responses to familiar and unknown faces were lower in institutionalized children, while Swingler et al. (2010) found ERP latencies to be associated with infant behavioral response to maternal separation. Mesquita et al. (2015) showed altered ERP components magnitude in response to faces in children with atypical social behaviors and recently (Kungl et al., 2017) found an association between attachment security and face brain responses. In addition, recent studies show that in healthy children the quality of the maternal environment is related to the magnitude of ERP components in response to emotional faces (Carlsson et al., 2008; Taylor-Colls and Pasco Fearon, 2015), confirming the association between early interactive experience and brain development of face perception. As a consequence, it is possible that an early dysfunction of the relevant circuitry of neural face processing could affect the quality of the interactions, and probably also decrease the quality of the child environment, contributing to activate a negative developmental pathway. Up to date limited information is available on how infants process and respond to social stimuli in early at-risk conditions. In particular, no study investigated whether early human face processing is susceptible to antenatal growth and/or might be affected by fetal growth restriction. Indeed, in the study of IUGR, researches are needed in order to ensure that facial processing is not compromised by their antenatal adversities slowing down the fetal growth. In fact, in the light of studies on clinical groups (Parker and Nelson, 2005; Nelson and McCleery, 2008), it appears as of highest clinical importance to understand the role of early adversities on neural face processing and how altered face processing could be conceived as early marched on possible risk on socio-emotional development.

With the aim of bridging the above-described research focuses and objectives, the present study protocol attempts to open a new research perspective on early development of IUGR infants, following their interactive and neural developmental pathways across the first year of life. By comparing IUGR with healthy children, we study the effect of antenatal adversity on brain functionality and interactive abilities. Specifically, aim of the study will be to investigate whether growth restriction significantly affect socio-cognitive developmental at 12 months both directly and thought the mediation of behavioral and neural response to social stimuli as displayed at 4 and 9 months. In particular, mediation hypotheses cover the following pathways:

- *Infant behavioral responsiveness in social interaction*: since studies on IUGR population support IUGR infants’ greater passivity, communicative difficulties in early mother-child exchanges and an early disinclination to be engaged by human faces, we investigate a group difference (IUGR—Control), expecting IUGR lower levels of responsivity to maternal stimulation during free-play exchanges. Worse communicative abilities in the IUGR group can lead to an impoverished environment for the infant and therefore fewer opportunities for stimulation and learning. In turn, this could affect the cognitive development and therefore could represent a mechanism linking the stress experienced during intrauterine life to later adaptation.
- *Infant neural social processing*: many evidences showed that neural competence in face processing is significantly altered in clinical populations, advocating a likely role of this neural domain in sustaining and worsening the effects of early stress on child development. Since research on IUGR show their difficulties in engaging with faces and social situations, we suggest a potential role for face processing neural correlates in the association between antenatal growth restriction and cognitive outcomes. Therefore, we aim to explore the role of the scalp-recorded cortical activity, in terms of ERPs, in response to social and non-social stimuli in IUGR and non IUGR infants. The following ERP components will be selected in accordance with current evidence of the literature on infants’ face processing—i.e., the infant N170 at around 290 ms after the stimulus onset and P400 (de Haan and Nelson, 1999; de Haan et al., 2003; Moulson et al., 2009), and emotional/attentional processing—i.e., Negative central (Nc; Moulson et al., 2009; Taylor-Colls and Pasco Fearon, 2015). Indeed, by considering several ERP components, the potential role of intrauterine growth adversity on social processing will be linked to specific features of neural processing. Specifically, we expect to find reduced amplitude (N170, Nc) and latency (P400) in the IUGR group. In addition, we hypothesized that these alterations in brain functionality in response to human faces can represent an early marker of the later cognitive deficit of IUGR infants, therefore suggesting a mediation effect.

Then, cortical response to social stimuli will be investigated as possible underpinnings of reduced responsiveness to maternal environment in IUGR infants, compared to matched healthy controls. Therefore, positive correlations will be expected, highlighting this link.

Last, along with the mediation roles expected for child behavioral and neural social responsivity, maternal environment, in terms of sensitivity, will be investigated at an explorative level as exerting a moderating role in the association between infant responsivity on cognitive and behavioral development assessed at 12th months. Indeed, although no specific evidence suggests that maternal sensitivity can modulate the developmental trajectories in IUGR samples, this effect has been shown in other at-risk population such as premature infants. Therefore, we aim at considering moderator effects in order to detect potential buffering or detrimental roles of maternal environment in infants experiencing fetal stress.

**Figure 1:**
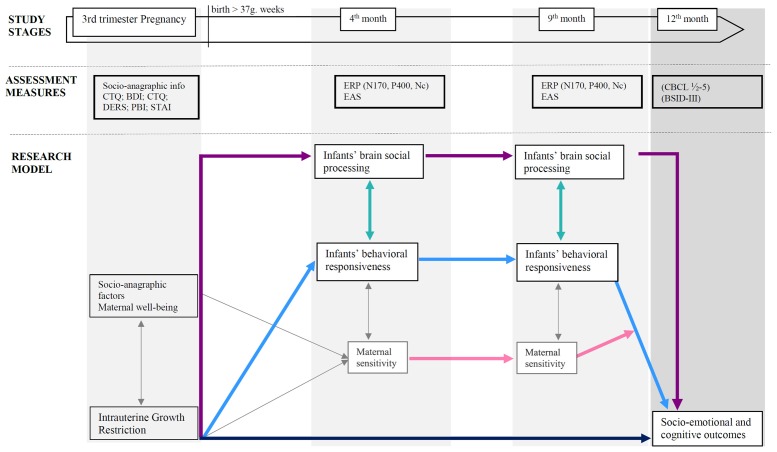
Research design and hypothesized associations between variables. Colored arrows depict research hypotheses; gray thin arrows refer to expected paths not considered in this study protocol for sake of brevity. *Note*: PBI, Parental Bonding Instrument (Parker, 1989); CTQ, Childhood Trauma Questionnaire (Bernstein et al., 2003); BDI-II, Beck Depression Inventory-II (Beck et al., 1996); STAI, State Trait Anxiety Inventory (Spielberger, 1983); DERS, Difficulties in Emotion Regulation (Scale Gratz and Roemer, 2004); ERPs, Event-related potentials, EASs, Emotional Availability Scales (Biringen, 2008); CBCL ½-5, Child Behavior Checklist ½-5 (Achenbach and Rescorla, 2000); BSID-III, Bayley Scales for Infant and Toddler Development—Third Version (Bayley, 2006).

Overall, research design and hypotheses are graphically summarized in Figure 1.

## Method

### Participants

For the IUGR group, 30 pregnant women will be recruited at the Department of Women’s and Child’s Health, University of Padova (Italy). Healthy control pregnant mothers (*N* = 30) will be recruited from birth-preparation courses of the Obstetrics and Gynecological Clinic of Padua Hospital. All pregnant women are orally presented with a longitudinal study on the role of IUGR on child socio-emotional development by a Gynecologist and a Psychologist, while waiting their visit or the birth-preparation class. For mothers with pregnancy complicated by IUGR; research will be proposed at the first obstetrical visit following diagnosis. All mothers interested in the study will receive a detailed informative module, describing stages and tasks on the study. In particular, they will be informed on the study length and procedures; absence of risk for both behavioral and EEG assessment is declared, and a potential tolerable level of discomfort is reported for the EEG cup wearing procedure. Participants will also be informed on the possibility to withdraw their participation at any time without giving an explanation and that their decision would not affect future healthcare encounters. In accordance with the Declaration of Helsinki, prior to first assessment, parents agreeing to be involved in the present study will sign written informed consent. Mothers will sign an informed consent as participants; while two other different consent forms are required to be signed by both parents for infant’s participation, namely in the behavioral and neuroimaging assessments. The present study received ethical approval from the Ethic Committee of the University of Padua (protocol reference number: 2293).

Participants will be mother-infant dyads who received *in utero* IUGR diagnosis (verified by Doppler ultrasound and estimated birth weight below the 10th percentile of growth), confirmed by birth weight below the 10th percentile of growth curve. Infants exclusion criteria will be: genetic disorders, unrelated comorbidities, presence of fetal infections, congenital malformations (i.e., congenital heart disease), metabolic and chromosomal disorders at birth, as well as infant neurological pathologies, brain abnormalities, or preterm delivery (<37th gestational week). Mothers’ exclusion criteria will be IUGR diagnosis before the 7th month of pregnancy and complicated pregnancies, non-Italian nationality, mother age <18 years, psychiatric disorders’ risk as defined by clinical score (namely a Global Symptom Index >65) in the Symptom Checklist 90-Revised (Derogatis, 1975), neurocognitive disorders, drug addiction, single mothers.

### Procedure

This project describes a longitudinal research, articulated in four stages over the first year of the child’s life. During recruitment at the pregnancy stage demographical information will be collected through a detailed paper-and-pencil evaluation. Indeed, an *ad hoc* socio-demographic assessment has been designed in order to collect comprehensive information about maternal age, cohabitation, marital status, education, work, parity and presence of previous abortion or at-risk pregnancies.

In addition, psycho-social and clinical-psychological status of the mother and of the whole family system will be assessed, by applying the following self-report questionnaires: Childhood Trauma Questionnaire (CTQ)—Short Form (Bernstein et al., 2003; Sacchi et al., 2017); Parental Bonding Instrument (PBI; Parker, 1989); Difficulties in Emotion Regulation Scale (DERS; Gratz and Roemer, 2004); State Trait Anxiety Inventory (STAI; Spielberger, 1983); Beck Depression Inventory-II (BDI-II Beck et al., 1996).

After recruitment and the first assessment during pregnancy taking place at the Hospital, all participants agreeing to take part into the study will be telephonically contacted at the 4th month of child’s life for behavioral and neuroimaging assessment. In particular, mother-child couples will be invited to visit the Department of Developmental Psychology and Socialization at the University of Padova. At 4 and 9 months, assessment procedure will involve free play interactions and EEG recording at the Inter-departmental High-density EEG lab; while at 12 months, developmental outcomes will be measured with children assessed on cognitive development by a structured procedure performed by trained psychologist, and emotional-behavioral problems rated by mothers.

#### Cognitive and Socio-Emotional Development at 12 Months of Life

Cognitive assessment will be performed at 12 months of child’s life using Bayley Scales for Infant Development—Third Version (BSID—III; Bayley, 2006); which evaluates five different domains: cognitive, language, motor, socio-emotional behavior and adaptive behavior. Evaluation of the first three domains consist of a direct observation of the child performance on different task, while socio-emotional and adaptive behaviors are parent rated. For direct assessment, each item is assessed on a dichotomous scale, with 1 given to child’s ability to perform the targeted behavior and 0 to the absence of such behavior. After five consecutive missing behaviors the scale’s administration is interrupted. Cognitive scale is composed by 91 items assessing: sensorimotor development, exploration and manipulation, object relatedness, concept formation, and memory. Language scale is composed by 49 items referring to receptive communication (i.e., pre-verbal behavior, vocabulary development, morphological development, understanding morphological markers, social referencing and verbal comprehension), and 48 items assessing pre-verbal communications (i.e., vocabulary development and morpho-syntactic development). Motor scale examines fine motor and gross motor domains. In particular, fine motor subtest is composed by 66 items about: prehension, perceptual-motor integration, motor planning and speed, visual tracking, reaching, object grasping, object manipulation, functional hand skills, responses to tactile information. Gross motor subtest refers to 72 items covering movement of the limbs and torso, static positioning (e.g., sitting, standing), dynamic movement (including locomotion and coordination), balance, and motor planning.

Socio-emotional scale represents and adaptation from the Greenspan social-emotional growth chart (Greenspan, 2004) assessing child self-regulation, communicating needs, the ability to establish relationship and the use of emotions for interactive purposes or to solve problems. Last, Adaptive Behavior assessment refers to child’s social, motor, pre-academics, home living, self-care, self-direction, community use, leisure, communication, health and safety skills.

Each of the 5 scales provide a raw score, and a scaled score (*M* = 10, *SD* = 3). For Cognitive, language and motor scales also allow to compute composite scores, referred to a mean value of 100 and a standard deviation of 15. Composite scores lower than 85 were considered as abnormal performances (Albers and Grieve, 2007). Examinations will be performed by a trained psychologist with enduring experience in the BSID-III.

Socio-emotional development will be parent rated via Child Behavior Checklist ½-5 (CBCL ½-5; Achenbach and Rescorla, 2000), a checklist of 113 questions, scored on a three-point Likert scale (0 = Not True, 1 = Somewhat or Sometimes True, 2 = Very True or Often True, based on the past 6 months). CBCL provides scores for eight syndromes, three broadband domains (Internalizing, Externalizing, and Total Problems), and six DSM-oriented scales. Although CBCL was originally designed for child assessment from 18 months of age, previous studies showed its good psychometrical properties with 12 months infant and encouraged its downward extension (Van Zeijl et al., 2006a,b; Ramchandani et al., 2013).

#### Child Responsiveness and Maternal Sensitivity

Mother-child interactions will be video-recorded during free-play interactive exchanges lasting about 15 min. At this purpose, a quiet and silent room will be equipped with a kid rug, pillows and age-appropriate toys; namely: rattles, puppies, and soft activity books for 4 months infants; pop-up surprise box, soft telephone, blocks box, activity book, and rock-a-stuck for 9 months. Mothers will be instructed to freely interact with their baby as they are used to do at home; they are kindly asked to remain within camera focus, unless their baby show signs of distress and need to calm them down.

Emotional Availability Scales (EASs; Biringen, 2008) will be applied to code interactive behaviors following the coding system of the EA Third Edition. EAS constitutes of four parental dimensions: adult sensitivity, adult structuring, adult non-intrusiveness, adult non-hostility; and two child scales: child responsiveness and child involvement. Each EA dimension produces score on a 7-point scale, where higher ratings stand for more optimal features. Values between 5 and 7 are representative of an emotionally available dyad and considered index of a healthy relationship. Scores around 4 indicate complicated emotional availability, that is behaviors that are appropriate in some ways but that are not optimal. Scores around 3 indicate less optimal aspects while the range between 1 and 2 concerns more problematic behaviors (Biringen, 2008). According to EAS Third Edition, the 6 scales can also be scored on seven subscales each; this allows to observe and detect specific behaviors composing the six macro-categories. Among the six dimensions, Adult sensitivity and Child responsiveness will be selected for the purposes of the present study. Indeed, maternal sensitivity represents an early indicator of the quality of infant’s postnatal social environment. Child responsiveness will be selected as behavioral correlates of child’s early responsiveness to social stimuli, investigated as cortical response. Video-recorded interactions will be coded by two independent judges, trained on the EAS system, who will be blind with respect to objectives and design of the study.

#### EEG Recording, Signal Processing and ERP Components Selection

Infants’ cortical activity will be continuously recorded using a Geodesic EEG system (EGI) through a pre-cabled high-density 128-channel HydroCel Geodesic Sensor Net (HCGSN-128) referenced to the vertex. While infants are placed on their mother’s legs in front of a screen at about 50 cm of distance, brain activity will be registered, through the use of the elastic sensor nets fitting each participant’s head size. Each electrode channel of the net is enveloped by a sponge and protected by a soft, plastic pedestal; this guarantee participants’ skin contact is only with sponge and plastic parts. Before assembly, Sensor Net is immersed in a shampoo, potassium chloride and distilled water solution for 5 min. After disassembly, all the non-disposable material used during the experiment (net, electrodes), is always disinfected before a subsequent re-use.

The electrophysiological data collection will last about 30 min per each infant, including equipment assembly and disassembly; also, to maximize infant comfort, skin pressure points and overturned sensors are checked before data acquisition, in accordance with EGI recommendations. While seating on mothers’ legs in the overshadowed room, both social and non-social stimuli will be presented. Specifically, the experimental paradigm employed will be adapted from a previous study (Mento and Valenza, 2016), and will involve the use of real female faces as social stimuli. Images of unfamiliar toys will be selected as the visual non-social stimuli. A total of 100 trials per condition will be delivered. During the procedure, infants’ behavior will be continuously monitored via a video camera, in order to allow the experimenter to decide when deliver on the screen attention-getter audio-visual stimuli (cartoon scenes) as soon as infants attention on the screen will be loose. The electrical signal will be filtered with a 0.1-Hz to 100-Hz band-pass with a sampling rate of 500 Hz.

Consistent with previous studies on face and emotion processing in infants (de Haan and Nelson, 1999; de Haan et al., 2003; Taylor-Colls and Pasco Fearon, 2015; Guy et al., 2016), component timings will be selected as follows: the infant N170 component will be selected as the early correlate of specialized face processing in infants (infant 170), reflecting structural features of face processing. This component has been consistently shown to exhibit greater amplitude in response to faces as compared to visual noise in 3 month-old infants (Halit et al., 2004) and also to familial vs. non-familial faces at 9 months (Scott et al., 2006). The infant N170 will be expected to peak negative in amplitude 290–350 ms after stimulus onset in posterior electrodes (de Haan et al., 2003). Second, the P400 will be considered as involved in high-order face processing; the P400 represents a positive component peaking between 390 ms and 450 ms after stimulus onset and maximal over occipital electrodes (de Haan et al., 2003). Last, the “Nc,” component will be considered as relevant components of late face-processing (de Haan et al., 2003). The Nc will be defined as the negative EEG deflection occurring between 350 ms and 750 ms after stimulus onset over frontal and central midline electrodes (Guy et al., 2016). The Nc component is thought to reflect the activation of attentional processing linked to the appraisal of the motivational significance of emotional expressions (Taylor-Colls and Pasco Fearon, 2015).

The EEG recordings will be processed offline using MATLAB toolboxes EEGLAB and ERPLAB. EEG signal will be segmented into epochs beginning 100 ms before stimulus onset and ending 800 ms after. Prior to epoching procedure, videos will be visually inspected off-line in order to reject EEG segments where participants did not look at the screen. In order to identify, reject or correct bad channels, artifacts, eye blinks and eye movements, the Independent Component Analyses (Stone, 2002) will be applied on individual epoched EEG dataset. As the last step, data will be averaged and re-referenced to average reference. Only participants showing a minimum of 30 artifact-free trials per condition will be included in the grand average.

## Data Analysis

To answer the first research question about the social stimuli processing in IUGR infants in terms of amplitude and latency of ERP components, analysis will involve repeated measure models, with group (IUGR vs. Controls) as between factor and developmental stage (4–9 months) and stimuli condition (social vs. non-social) as within factors. No previous study is available to obtain an estimate of the target effect size; however, we can refer to Parker and Nelson (2005) work on institutionalized children compared with non-institutionalized children to obtain an estimation of the effect of clinical conditions on ERP components in response to human faces. Even if it is unlikely that a perinatal condition such being IUGR is comparable with a complex relational stressor as being raised in an institution, this study can provide a rough estimation of the effect involved in the present protocol. Indeed, they found differences in N170, Nc, PSW and P250 amplitude between groups that range from intermediate to large. In the present study, considering the planned sample size, we should be able to obtain a 0.98 power to detect a small effect (repeated measures ANOVA within-between interaction, G*Power 3.1.9.2, Faul et al., 2009), which seems satisfactory in relation to the previous findings.

Second, to test the mediation effect of both neural response to social stimuli and behavioral child responsiveness on cognitive and neurodevelopment outcomes, Hayes approach will be followed (Hayes, 2013). The power to detect a direct an intermediate effect of IUGR condition on 12 months outcome is above 0.80 (difference between two independent means, G*Power 3.1.9.2). For what concerns indirect effects, mediation models have usually larger effect sizes than main effects (Kenny and Judd, 2014). Last, at a more explorative level a path analysis will be conducted to study the moderation role of maternal sensitivity in the previous mediation models. In particular, the moderation effect on the direct association between IUGR condition and later outcome as well as on the association between IUGR condition and child responsiveness will be explored.

## Expected Results

For the developmental outcomes at 12 months, in line with previous studies (Fernandez-Carrocera et al., 2003; Batalle et al., 2013), we expect poorer cognitive and behavioral performances in IUGR infants, compared to control peers, as result of both a direct effect of being IUGR and a mediation of neural and behavioral responding to social stimuli.

On the behavioral domain, lower levels of child responsiveness during mother-child interactions are expected within the IUGR group, evidencing poorer behavioral responses to social stimuli, in accordance with evidence of IUGR greater passivity during social exchanges (Feldman and Eidelman, 2006). Then, significant positive correlations between ERPs amplitude for social stimuli and behavioral responsiveness to maternal stimulations are expected across groups, suggesting that early face processing might be conceived as a neural correlate of child responsiveness during mother-child interactive exchanges.

As regards the investigation of the neural mechanisms sustaining infants processing of social stimuli, temporal resolution given by the application of the EEG will allow to test the potential effect of being IUGR on different steps of face processing. Specifically, differences in infant N170 amplitude will allow to detect a potential role of being IUGR on basic structural features of face processing, while differences in P400 latency between groups, expected in the direction of longer latency for IUGR performances, will allow to detect an atypical IUGR processing regarding more complex steps of face processing. Last, between-groups difference in the Nc component will be tested in order to highlight atypical attention engagement in IUGR infants.

Considering in details the potential differences in ERP components in response to social and non-social conditions, we first expect faces to elicit greater amplitude in infant N170 and Nc and shorter latency in P400 than toys across groups (IUGR vs. Controls), in line with previous studies on infants face processing (Taylor-Colls and Pasco Fearon, 2015; Guy et al., 2016). Second, we expect that the prenatal stress experienced by IUGR infants results in smaller ERPs amplitude for social stimuli in the IUGR group, similarly to other at risk populations exposed to early adverse conditions, such as institutionalized children and young children with autism (Nelson and McCleery, 2008). Third, we expect an interaction effect Group × Condition, resulting in a reduced difference in amplitude between the social vs. non-social conditions for the IUGR group. Moreover, at an explorative level, the longitudinal design of the study will offer the opportunity to investigate whether neural social processing is susceptible to different pathways of specialization across groups (IUGR vs. Controls), as displayed by potential between-groups differences in neural face responses across steps (4 vs. 9 months). No specific results are expected, but a tendency toward stability across stages of the hypothesized detrimental effect of prenatal stress on neural face processing would suggest the presence of an atypical developmental trajectory for the IUGR population. On the contrary, a tendency toward a decreasing gap between groups would point toward considering face processing in IUGR as a stage-dependent mechanism limited in time, even if the potentially negative effect on long term outcomes could remain.

However, the limited knowledge in the functionality of IUGR brain in response to social stimuli does not ensure that group differences can be found in the hypothesized components or that they are located in the same brain regions of typically developed children. In this respect, subsequent exploratory analyses can enrich the quality of the investigation by means of data driven approach able to study the overall brain functionality (i.e., Maris, 2004).

Last, about the role of infants’ postnatal environment, high maternal sensitivity, considered as a proxy of the overall maternal environment quality, is expected to buffer the effect of adversities in fetal growth on later developmental outcome by enhancing child’s engagement and responsivity to social environment.

## Discussion

Recent approaches to the study of early brain development are shifting backward sensitive epochs, emphasizing the role of antenatal life and fetal growth. Framed in this context, Barker’s (1998) hypothesis of fetal programming suggests that adverse influences during intrauterine life, such as growth restriction, can result in permanent long-term changes in physiology and metabolism, increasing the risk for adult diseases and health problems. The present study pursues the objective of broadening this research field providing new insights on the interconnected role of both antenatal and postnatal life on cognitive and emotional-behavioral development. In particular, results deriving from this research project will enhance understanding on early neural mechanisms underpinning the interactive-relational patterns sustaining socio-cognitive development in infants with IUGR. Indeed, this study represents a first contribution to understand whether antenatal stress in terms of fetal growth delay is likely to affect early neural competences of face processing and whether this capacity represents a neural correlate of altered behavioral-interactive development along the first year. In particular, the evidence of a role of intrauterine life experiences in affecting later face processing would enhance our understanding of the development of this fundamental ability and its experience-expectant and activity-dependent nature. In addition, the study will also help in clarifying the role of (prenatal and postnatal) mother-child exchanges in buffering the vulnerability experienced by children delayed in their fetal growth. Indeed, even if it is difficult to disentangle the direction of the effects, it is clinically relevant considering infant’s face processing and environmental quality in the study of developmental trajectories of children experiencing early adversities, such as alterations in the antenatal growth. The present study also aims to develop the perspective proposed by Taylor-Colls and Pasco Fearon (2015) on the role of parental quality on infants’ neural response to emotional faces, in which further studies on clinical and at-risk populations and longitudinal designs are claimed. However, in the study of IUGR, before considering the neural response to emotional cues, a step back is needed in order to ensure that facial processing is not compromised by the antenatal adversities that slow down the fetal growth. Therefore, the present study will provide a preliminary link, opening the way for further studies on early social processing in IUGR infants.

As a first attempt in the study of IUGR socio-emotional fragility, our protocol still presents some potential limitations. First, the aforementioned lack of knowledge on the specificity of brain functionality of IUGR infants does not ensure that ERP components can be found with the same localization and characteristics to be compared with typically developing children. Second, the selection criteria of excluding IUGR infants born before the 37 gestational week ensures a specific focus on the unique role of being IUGR as a source of antenatal stress, apart from the stress and physical pain experienced by premature infants after birth (Montirosso et al., 2018). However, future studies could explore potential differences between term IUGR and preterm IUGR, in order to disentangle the specific contribution of ante and post-natal stress in infant development. Third, IUGR disorder could result associated with highly severe maternal conditions during pregnancy such as infections, toxins, prescriptions drugs, substances abuse, that affect both intrauterine and postnatal environment (Brancato and Cannizzaro, 2018). In the present protocol, severe maternal conditions were excluded in order to study the specific effect of being IUGR, but future investigations with a similar methodology could consider whether IUGR is one of the mechanisms involved in the child detrimental outcomes of these maternal conditions.

## Conclusion

In conclusion, very few is known on the effect of antenatal growth on socio-emotional development during early infancy. Studies investigating early pattern of social processing (Tronick and Beeghly, 2011), both in terms of neural and behavioral features in clinical or at-risk groups, have the potential to early inform on underpinning mechanisms exposing vulnerable infants to different developmental pathways (Fumagalli et al., 2018). Overall, the clinical relevance of the present research protocol lays in designing a longitudinal research perspective where, despite the laboratory setting, the selected tasks rely on processes (i.e., face perception and mother child interactions) relatively ecological for infants. More importantly, infants neural and behavioral competences are combined to at least partially switch on a light on one of the potential pathways through which antenatal adversities translate into development fragilities, before fragility becomes a clinical outcome. Addressing this question has the clinical relevance to translate results into applicative guidelines in order to potentially generate effective and empirically-driven interventions in early infancy. Indeed, considering possible difficulties of IUGR infants in face processing and behavioral interactions might help in developing early *ad hoc* interventions aimed at supporting mothers in sensitive and multimodal communications, thus hopefully constraining the effect of infant’s social processing deficits on later socio-emotional development. Last, a second-order implication of the present protocol is that it might be generalizable to several developmental risks’ population deriving from decreased or altered antenatal growth trajectories (i.e., prematurity, congenital heart disease; maternal substance abuse) in order to identify differential trajectories starting form specific etiopathological conditions, or rather common mechanisms predisposing to multiple outcomes.

## Author Contibutions

CS designed the study and drafted the manuscript. PDC contributed to the manuscript draft, planned the analyses, and performed the power analysis. GM designed the experimental tasks and revised the manuscript critically for important intellectual content. TF contributed to the design of the experimental tasks and revised the manuscript critically for important intellectual content. SV contributed to the study design. AS mentored the first author in designing the study, revised the manuscript critically for important intellectual content. All the authors carefully read and approved the final version of the manuscript.

## Conflict of Interest Statement

The authors declare that the research was conducted in the absence of any commercial or financial relationships that could be construed as a potential conflict of interest.
